# Evaluation of an automated safety surveillance system using risk adjusted sequential probability ratio testing

**DOI:** 10.1186/1472-6947-11-75

**Published:** 2011-12-14

**Authors:** Michael E Matheny, Sharon-Lise T Normand, Thomas P Gross, Danica Marinac-Dabic, Nilsa Loyo-Berrios, Venkatesan D Vidi, Sharon Donnelly, Frederic S Resnic

**Affiliations:** 1 GRECC and Center for Health Services Research, Tennessee Valley Healthcare System, Veterans Administration, Nashville, TN, USA; 2 Division of General Internal Medicine and Public Health, Vanderbilt University Medical Center, Nashville, TN, USA; 3 Department of Biomedical Informatics, Vanderbilt University Medical Center, Nashville, TN, USA; 4 Department of Health Care Policy, Harvard Medical School and Department of Biostatistics, Harvard School of Public Health, Boston, MA, USA; 5 Food and Drug Administration, Center for Devices and Radiological Health, Silver Spring, MD, USA; 6 Division of Cardiology, Brigham & Women's Hospital, Boston, MA, USA

## Abstract

**Background:**

Automated adverse outcome surveillance tools and methods have potential utility in quality improvement and medical product surveillance activities. Their use for assessing hospital performance on the basis of patient outcomes has received little attention. We compared risk-adjusted sequential probability ratio testing (RA-SPRT) implemented in an automated tool to Massachusetts public reports of 30-day mortality after isolated coronary artery bypass graft surgery.

**Methods:**

A total of 23,020 isolated adult coronary artery bypass surgery admissions performed in Massachusetts hospitals between January 1, 2002 and September 30, 2007 were retrospectively re-evaluated. The RA-SPRT method was implemented within an automated surveillance tool to identify hospital outliers in yearly increments. We used an overall type I error rate of 0.05, an overall type II error rate of 0.10, and a threshold that signaled if the odds of dying 30-days after surgery was at least twice than expected. Annual hospital outlier status, based on the state-reported classification, was considered the gold standard. An event was defined as at least one occurrence of a higher-than-expected hospital mortality rate during a given year.

**Results:**

We examined a total of 83 hospital-year observations. The RA-SPRT method alerted 6 events among three hospitals for 30-day mortality compared with 5 events among two hospitals using the state public reports, yielding a sensitivity of 100% (5/5) and specificity of 98.8% (79/80).

**Conclusions:**

The automated RA-SPRT method performed well, detecting all of the true institutional outliers with a small false positive alerting rate. Such a system could provide confidential automated notification to local institutions in advance of public reporting providing opportunities for earlier quality improvement interventions.

## Background

Public reporting of risk adjusted mortality rates following cardiac surgery has become an important tool in the evaluation and improvement of quality of patient care [1]. Several states, including Massachusetts, have enacted legislation requiring public reporting of cardiac surgical outcome data [2]. Beginning in 2002, all Massachusetts hospitals performing cardiac surgery were required to submit cardiac surgical outcomes data to the Massachusetts Data Analysis Center (Mass-DAC), a data coordinating center for the Massachusetts Department of Public Health (MA DPH), and to the Society of Thoracic Surgeons (STS) using the STS National Cardiac Database collection tool. Since then, Mass-DAC has published annual public reports on 30-day, all-cause, risk-adjusted mortality rates for isolated coronary artery bypass surgery (CABG) for institutions, and beginning in 2004, for individual cardiac surgeons [3].

Though public reports are intended to provide transparency and public accountability, and to inform consumer choice, there are other consequences to public reporting that have potentially significant long-term, financial and reputational impact on both the institutions and providers. In Massachusetts, two centers were identified as statistically significant high mortality outliers, one of which was identified as such in multiple, consecutive years [4,5]. As a result, this cardiac surgery program was temporarily suspended while quality improvement initiatives were undertaken [6]. Notification of the first year of outlier status for this program was not available publicly until 2 to 2.75 years after data collection (relative to calendar quarter) [5,6].

There are inherent delays between performance of procedures and public reporting of outcomes due to the rigorous data review and manual case adjudication required from both a regulatory and data quality standpoint. Massachusetts has steadily decreased the delay between data collection and public reporting since the program's inception in 2002, currently reduced for fiscal year 2008 to 1.25 to 2 years (relative to calendar quarter) [7]. However, any temporal delays between the performance of the procedure and the analysis and public reporting of results is undesirable and could expose some patients to increased risk of morbidity and mortality as procedures continue to be performed while a quality issue exists. An active, automated prospective surveillance system, as an adjunct to the existing rigorous regulatory approach, could provide institutions or physicians timely internal feedback and provide opportunities to mitigate these risks well in advance of public release of the data.

We designed the Data Extraction and Longitudinal Trend Analysis (DELTA) system to provide real-time monitoring of clinical data to support continuous quality or safety monitoring of newly approved medical devices, medications, or therapeutic interventions [8]. This system supports a variety of frequentist and Bayesian statistical methods, which can be configured to provide unadjusted and risk-adjusted safety monitoring among prospective and retrospective cohorts [8-11]. DELTA also incorporates de-identification and encryption algorithms to guard protected health information and control data ownership, and employs flexible alerting mechanisms to trigger notifications via e-mail or through the web interface when an observed event rate exceeds boundaries of risk-adjusted expectations for the event of interest.

Risk-adjusted SPRT, a method for observational cohort safety surveillance, was first proposed by Spiegelhalter and colleagues [12]. This method has been used to evaluate hospital and physician performance among retrospective cohorts for coronary artery bypass patients and percutaneous coronary intervention patients [12-14]. While this method has not achieved widespread use, it is well suited for implementation in a prospective, automated system because it analyzes each sequential case and incorporates adjustment for repeated measures on the same subject with explicit type I and II error rates.

In this study, we sought to assess the utility of Risk-Adjusted Sequential Probability Ratio testing when imbedded in an automated surveillance tool as compared to the gold standard of retrospective annual quality reports used by the Massachusetts Department of Public Health (MA-DPH). The primary outcomes of this analysis were the sensitivity and specificity of the automated implementation as compared with the public reporting methods of Massachusetts Data Analysis Center (MASS-DAC) and the MA-DPH, assessed for hospital 30-day mortality after isolated coronary artery bypass graft surgery.

## Methods

### Study Setting

Massachusetts regulations require all acute care non-federal hospitals that provide cardiac surgery to collect data using a standardized data collection instrument based on the Society of Thoracic Surgery (STS) registry [15]. Each institution is required to submit data on a quarterly basis to Mass-DAC, and participating centers collect the data using a variety of point-of-care collection tools, chart review, and patient follow-up. Mass-DAC performs manual adjudication of all cases with adverse outcomes as well as a sample of all other case records. Yearly reports of hospital and surgeon 30-day mortality performance are published. Additional information and annual public reports are available online [3].

A total of 23,020 isolated adult coronary artery bypass surgery admissions were conducted from January 1, 2002 to September 30, 2007. The surgeries did not involve valve replacement or other associated cardiac surgical procedures. We selected these surgeries for our study because the state uses them as the primary index of institution and surgeon quality for cardiovascular surgery. In 2006, Mass-DAC changed reporting from a calendar year basis to a fiscal year basis that runs from October 1 through September 30. Consequently, the 2006 fiscal year analysis included the last three months of the 2005 calendar year. The primary patient outcome of the registry is the 30-day all-cause mortality after isolated coronary artery bypass graft surgery. We focused on 30-day all-cause hospital-specific risk-standardized mortality rates. The current study was approved by the Brigham & Women's and Harvard Medical School's Institutional Review Boards.

### Gold Standard Statistical Analysis

Mass-DAC reports the data annually utilizing Bayesian hierarchical logistic regression [1]. The model assumes that the log-odds of mortality is linearly related to a set of patient risk factors and permits baseline risk to vary across hospitals through the inclusion of a hospital-specific intercept. Estimation of the model parameters, including the between-hospital variance, hospital overall mean log-odds, and regression coefficients of patient-level risk factors are obtained via Markov chain Monte Carlo (MCMC) methods. The MCMC method uses the Gibbs sampler to sequentially sample from probability distributions and produces a Markov chain with the joint posterior density as its stationary distribution [16]. This is accomplished by selecting a set of starting values, then performing a number of "burn-in" sampling iterations that are not recorded, followed by the collection and averaging of additional sampling iterations to form the final posterior estimates. The primary analysis used all of the data for the year and declared a hospital as an outlier if the lower limit of the 95% posterior interval of the risk-adjusted institutional standardized mortality rate exceeded the unadjusted statewide mortality rate. Because of the small number of cardiac surgery hospitals in Massachusetts, Mass-DAC also performs cross-validation analyses in which each hospital is eliminated and data from the remaining hospitals are used to assess hospital performance in the eliminated hospital. This strategy was developed to avoid one large center from having too great an influence on statewide risk expectations. A hospital was considered an outlier if either the 95% posterior interval from the statewide comparison exceeded the unadjusted statewide mortality rate or the posterior predictive p-value from the cross-validation analysis was 0.01 or smaller.

**Figure 1:**
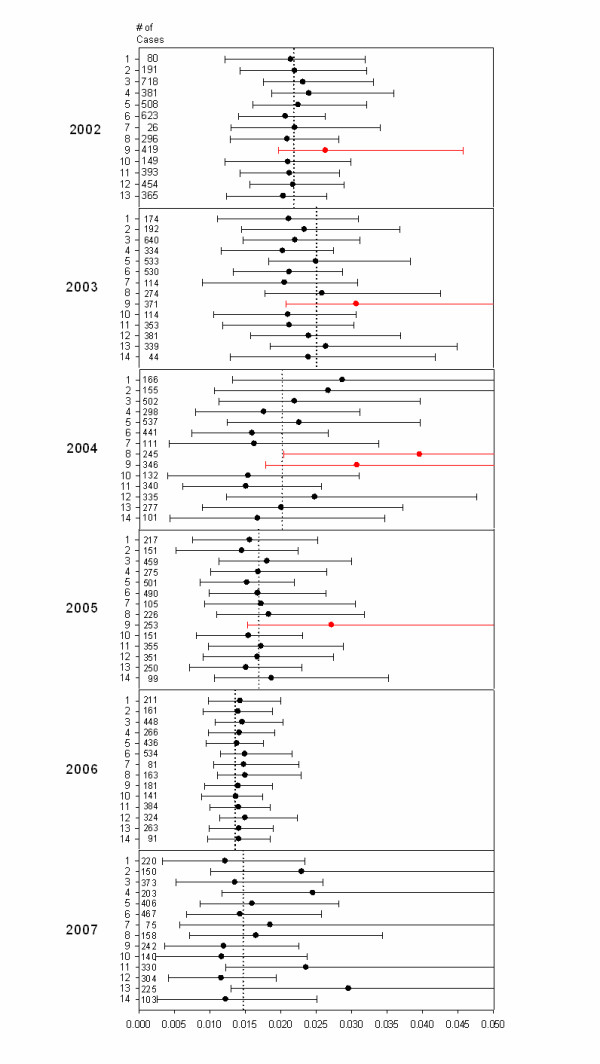
**Gold Standard Results for MASSDAC CABG by year**. Each number on the y axis within each year represents a unique hospital. Circles represent posterior mean risk-standardized mortality rates for each institution; lines are corresponding 95% posterior intervals. The hospitals in red alerted from the cross-validation method only.

Hospital 9 was declared an outlier in the 2002-2005 reports, and hospital 8 was declared an outlier in the 2004 report. All of hospital outliers were detected with only the cross-validation evaluation by MASS-DAC. Although the original 2002 public report did not include the cross-validation analysis method, we have exactly reproduced this analysis protocol on this data to provide consistency across all years. A summary of the risk-adjusted standardized mortality incidence rate (with upper and lower limits of the 95% interval) by hospital and year are reported in Figure 1. The dotted line in the figure represents the statewide unadjusted mortality incidence rate, and values in red indicated an outlying hospital.

### Automated Risk Adjusted Sequential Probability Ratio Testing (RA-SPRT)

The SPRT control chart methodology detects unacceptable event rates by evaluating each unit of analysis sequentially in time [12,13]. The risk hypothesis is whether the observed outcome event rate exceeds the accepted or baseline event rate given a specific odds ratio (OR) and Type I and II error [12]. This method accepts or rejects this hypothesis after each sequential case is evaluated. Risk adjustment is performed through the use of a risk prediction model whereby the cumulative log likelihood ratio is adjusted by the probability of the outcome [17]. Repeated measurement (re-analysis after each additional case) error adjustments are incorporated explicitly in the framework. These features are uncommon in statistical process control methods and are strengths of this method.

The following describes the calculations necessary to construct risk-adjusted SPRT control charts as refined by Rogers and colleagues[13] based on Spiegelhalter's work [12]. The control limits are defined by

(1) $$h_{0}=\frac{\ln\left(\frac{1-\alpha }{\beta }\right)}{\ln\left(OR\right)}$$

and

(2) $$h_{1}=\frac{\ln\left(\frac{1-\beta }{\alpha }\right)}{\ln\left(OR\right)}$$

where h_0 _and h_1 _are the cumulative log-likelihood ratio values in which the null hypothesis (H_0_) or the alternate hypothesis (H_1_) is accepted (respectively), OR is the odds ratio, α is the type I error rate, and β is the type II error rate.

The cumulative log-likelihood ratio value (T^cum^) is calculated in sequence for higher risk detection (OR > 1) with

(3) $$T_{i}^{Cum}=T_{i-1}^{Cum}+\left(O_{i}-s_{i}\right)$$

where T_0_^cum ^= 0, O_i _is the observed outcome (0 or 1) for a binary procedure for i^th ^case, and

(4) $$s_{i}=\frac{\ln\left(\left(1-p_{i}\right)+\left(OR*p_{i}\right)\right)}{\ln\left(OR\right)}$$

where p_i _is the calculated probability of the outcome for the i^th ^case as determined by the risk prediction model.

We have previously described an automated real-time safety monitoring tool, Data Extraction and Longitudinal Trend Analysis (DELTA), that is able to perform larger numbers of concurrent prospective analyses using a variety of statistical methodologies and alerting thresholds [18]. The system uses a SQL 2005 server (Microsoft Corp., Redmond, WA) to provide internal data storage and configuration information, as well as providing the capability to integrate with external databases. The user interface was developed in the Microsoft. NET programming environment and was displayed in a web browser from a Microsoft IIS 6.0 Web Server (Microsoft Corp., Redmond, WA). Security of patient data is further addressed by record de-identification steps and user login access restrictions [19].

The RA-SPRT method was implemented directly within DELTA, and the statistical module evaluated the data after setting cohort inclusion and exclusion criteria as well as the necessary statistical parameters, such as the desired odds ratio and the type I and II error. Parameters and risk variable selection for the logistic regression risk adjustment were then passed through a bi-directional interface to SAS (Version 9.1, Cary, NC) in order to develop the required logistic regression models.

### Statistical Analysis

The risk-adjusted sequential probability ratio testing (RA-SPRT) method was used to evaluate the data separately for each calendar or fiscal year. Although one of the strengths of this method is that it can accumulate data continuously until the alerting odds ratio hypothesis is accepted or rejected, analyses were terminated at the end of each calendar or fiscal year and the cumulative log-likelihood ratio was reset to 0. This was done in order to be directly comparable to the gold standard. Risk adjustment was performed using standard logistic regression with the same risk factors used in the source method by Mass-DAC. Each logistic regression model was developed from data in the prior 11 months and then applied sequentially to each case in the "current" month. This process was repeated throughout the entire range of the data. Data were not available prior to 2002, so the models developed prior to December 2002 used from one to ten months of data (depending on the analysis month). Data from January 2002 were not analyzed by RA-SPRT because they were required to build the first model. It should be noted that the risk models developed from the first two months of data showed regression coefficient instability and poor calibration, as would be expected with low sample sizes. Both of these measurements subsequently stabilized for the remainder of the data. A type I error of 0.05 and type II error level of 0.10 were used in each of the RA-SPRT analyses, and an OR threshold of 2.0 was defined as the reasonable thresholds for concern regarding the clinical quality of the institution evaluated. An outlier was declared if a hospital exceeded the log likelihood ratio threshold at any point during that calendar year.

## Results

**Figure 2:**
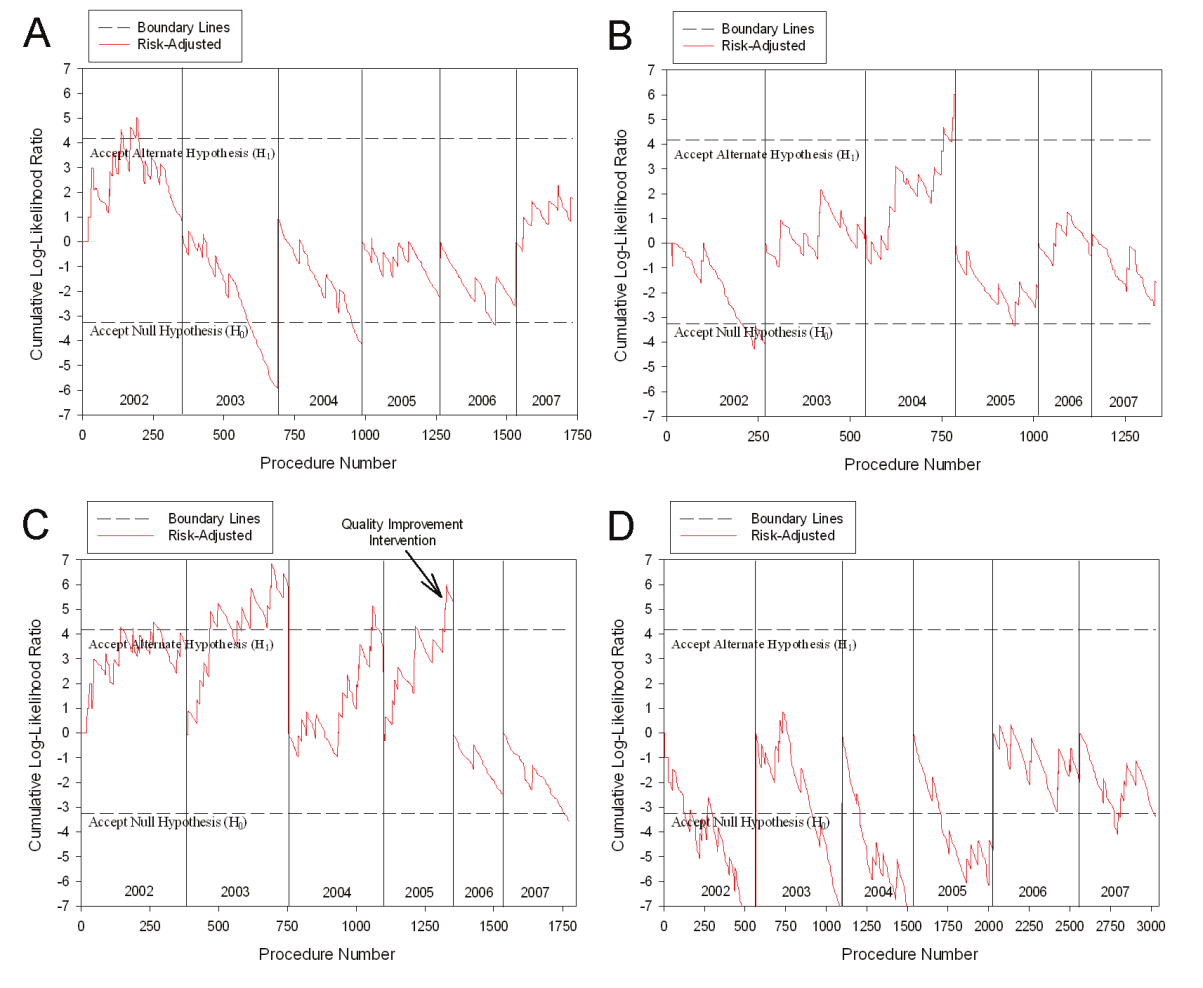
**Yearly RA-SPRT results (OR 2.0) for selected hospitals in which the graph was reset at the beginning of each year**. The upper threshold represents the alternate hypothesis (H_1_) confirming the odds ratio for the outcome ≥ 2.0, and the lower threshold represents the null hypothesis (H_0_) there was not an elevated odds ratio for the outcome ≥ 2.0. Years are calendar years except 2006-2007, which were fiscal years October - September. A) Hospital 4, B) Hospital 8, C) Hospital 9, D) Hospital 6.

A total of 23,020 isolated coronary artery bypass graft surgeries were evaluated from January 1^st^, 2002 to September 30^th^, 2007, and 925 cases in the last quarter of 2005 were included in both calendar year 2005 and fiscal year 2006 evaluations to be consistent with MASS-DAC reporting. The annual 30-day mortality rate ranged from 1.41 percent to 2.19 percent with a general decreasing trend. Most of the risk factors included in the risk models were stable across years, with the exceptions of declining prior CABG surgeries, increasing prior percutaneous coronary transluminal angiography procedures, and declining cardiogenic shock rates. A summary of patient risk factor prevalence and outcome event rates by year are shown in Table 1. The risk-adjusted SPRT method detected three hospitals with outlying 30-day mortality outcomes. Hospital 4 experienced a false positive alert in 2002 (Figure 2A), Hospital 8 experienced a true positive alert in 2004 (Figure 2B), and Hospital 9 experienced true positive alerts in 2002, 2003, 2004, and 2005 (Figure 2C). The remaining evaluations for each hospital were true negatives (for example, Hospital 6 shown in Figure 2D). This resulted in a sensitivity of RA-SPRT of 100% (5/5) and the specificity was 98.8% (79/80) compared to the publicly available reports.

**Table 1:** Summary information for patient and institutions by year.

	2002	2003	2004	2005	2006	2007
Number of Hospitals (count)	13	14	14	14	14	14
Number of Admissions (count)	4604	4393	3986	3885	3684	3396
30-Day Crude Mortality	2.17	2.25	2.01	1.65	1.41	1.47
	(1.77-2.63)	(1.84-2.74)	(1.60-2.49)	(1.27-2.10)	(1.06-1.85)	(1.09-1.94)

Mean Age in Years (SD)	66.5	66.7	66.9	66.5	66.5	66.0
	(10.7)	(10.6)	(10.7)	(10.8)	(10.7)	(10.9)
Male	74.5	73.5	74.5	76.4	75.2	75.9
	(73.2-75.8)	(72.2-74.8)	(73.1-75.9)	(75.1-77.8)	(73.8-76.7)	(74.4-77.3)
Renal Failure	7.3	6.9	5.8	6.4	6.6	7.5
	(6.6-8.1)	(6.2-7.7)	(5.1-6.6)	(5.6-7.2)	(5.8-7.4)	(6.6-8.4)
Diabetes Mellitus	38.0	38.1	37.0	39.3	39.4	42.6
	(36.6-39.4)	(36.6-39.5)	(35.5-38.5)	(37.8-40.8)	(37.9-41.0)	(40.9-44.3)
Hypertension	77.0	79.5	82.7	83.9	84.1	83.5
	(75.7-78.2)	(78.3-80.7)	(81.1-83.5)	(82.7-85.1)	(82.9-85.3)	(82.2-84.8)
Peripheral Vascular Disease	18.0	17.4	17.7	17.4	17.4	17.4
	(16.9-19.2)	(16.3-18.5)	(16.5-18.9)	(16.3-18.7)	(16.1-18.6)	(16.1-18.7)
Prior CABG Surgery	3.8	3.1	2.6	2.5	1.8	2.1
	(3.3-4.4)	(2.6-3.7)	(2.1-3.2)	(2.0-3.0)	(1.4-2.3)	(1.6-2.6)
Prior PTCA/PCI	18.6	17.8	19.1	20.2	21.0	21.6
	(17.5-19.8)	(16.7-18.9)	(17.8-20.3)	(19.0-21.6)	(19.7-22.3)	(20.2-23.0)
Cardiogenic Shock	2.2	1.6	1.1	1.0	0.9	0.8
	(1.8-2.7)	(1.3-2.0)	(0.8-1.5)	(0.7-1.4)	(0.6-1.2)	(0.5-1.1)
Ejection Fraction						
<30% or missing	12.8	12.6	11.8	11.6	10.1	10.4
	(11.9-13.9)	(11.4-13.4)	(10.8-12.8)	(10.6-12.7)	(9.1-11.1)	(9.5-11.6)
30 - 39	11.7	12.4	11.0	10.9	10.0	10.5
	(10.8-12.7)	(11.4-13.4)	(10.1-12.0)	(9.9-11.9)	(9.0-11.0)	(9.5-11.6)
Myocardial Infarction						
Within 24 Hours	2.7	3.2	3.8	2.9	3.3	3.3
	(2.3-3.2)	(2.6-3.7)	(3.3-4.5)	(2.4-3.5)	(2.7-3.9)	(2.7-4.0)
1 - 7 days	20.7	23.0	22.1	23.1	23.4	24.5
	(19.5-21.9)	(21.7-24.2)	(20.8-23.4)	(21.8-24.4)	(22.0-24.8)	(23.1-26.0)
Status of CABG Surgery						
Urgent	62.0	65.8	66.6	61.3	59.6	62.3
	(60.1-63.4)	(64.3-37.2)	(65.1-68.0)	(59.8-62.8)	(58.0-61.2)	(60.7-64.0)
Emergent/Salvage	3.7	2.9	3.1	2.5	2.6	3.3
	((3.2-4.3)	(2.4-3.4)	(2.6-3.7)	(2.0-3.0)	(2.1-3.1)	(2.7-3.9)
Pre-op Intra-Aortic Balloon Pump	9.3	11.7	11.6	10.9	11.2	11.3
	(8.5-10.2)	(10.7-12.6)	(10.6-12.6)	(9.9-11.9)	(10.2-12.3)	(10.2-12.4)

Years 2002-2005 were collected in the calendar year, and 2006-2007 were collected in the fiscal year from October of the previous year to September of the reported year. All data are percents (with 95% confidence intervals) unless otherwise specified as means (with standard deviations).

## Discussion

The automated RA-SPRT method within DELTA performed well applied to a statewide clinical registry data over a number of years when compared with the method used by the state to produce the public reporting of institutional and physician performance reports. The method detected each of the true outliers and generated a single false positive, which is a desirable profile for an early detection system used for knowledge discovery and internal quality improvement initiatives. Due to the frequent periodic analysis that the method employs, the DELTA system would have reported these findings to the local institutions significantly earlier than they were available publicly.

In general, an automated outcomes surveillance system, whether used for post-marketing surveillance or institutional and physician profiling, should be tuned with appropriate error levels and alerting thresholds to be over-sensitive in a manner similar to a screening test, where it is highly desirable to capture all of the true signals and tolerance to false positive signals is dependent on the resources that are available to perform root cause analyses and further data exploration. The RA-SPRT method did perform in this manner in this data set using stock values for types I and II error rates, and a common odds ratio threshold, which is encouraging, but does require further validations in other clinical domains to increase the generalizability of these findings. Selection of a desirable false positive versus false negative threshold depends on the clinical domain's desire to avoid missing a true signal and the cost of performing more in depth analysis of each detected signal. In addition, processing time was very reasonable within the automated application, and a quarter of data was able to be analyzed in seconds, with the most time consuming step being that of logistic regression model generation in each month analyzed.

The RA-SPRT method has a number of strengths, including explicit alerting boundary thresholds and incorporation of repeated testing [12-14]. It should be noted that the RA-SPRT method uses a simple hypothesis rather than a composite hypothesis that tests all odds ratios greater than 1.0 for statistical significance. This could potentially result in a statistically significant odds ratio less than the selected threshold (such as 1.1 or 1.2) that is not detected as an outlier by the method. However, in many cases, such an alert may not be clinically significant. In this study, we chose an odds ratio threshold that would be clearly clinically significant.

There are a number of limitations to this work. It should be noted that the sensitivity and specificity are optimistic in that implementation of this system in a prospective setting would eliminate the time costly manual data adjudication and outcome chart review steps that are done by Mass-DAC, at the expense of the accuracy of the data analyzed. Another limitation in the use of this system is the timing of data submission by the sites. Currently, data are submitted quarterly, rather than monthly (as used in this analysis), by MA cardiac institutions. The RA-SPRT method is intended for sequential case level analysis and performing the analysis in larger time segments results in over-correction of the repeated measurement adjustment. Real-time data surveillance would be ideal, but requires a regular and relatively clean data stream submission from the source institution. In order to evaluate this potential and evaluate the impact of lack of data adjudication, we are currently conducting percutaneous coronary angiography prospective surveillance in a subset of institutions in the state with the necessary infrastructure for near real-time submission.

## Conclusions

The RA-SPRT method implemented within an automated surveillance system was able to detect institutional outliers in a statewide clinical registry. While either a significant electronic health record infrastructure or a state reporting mechanism is required to realize the full utility of this system for outcome profiling, it could result in significant time savings in providing early warnings to local institutions and physicians.

## Competing interests

A patent application by Frederic Resnic, Michael Matheny, and Richard Cope for several key analytic components of DELTA is currently under review at the U.S. Patent Office. As current and former employees of Brigham & Women's Hospital, the intellectual property rights of Frederic Resnic and Michael Matheny related to the development of DELTA are assigned to the institution. Neither Frederic Resnic nor Michael Matheny have previously received nor anticipate any financial compensation, stock options, income, or ownership in DELTA, Coping Systems, or any other related commercial entity. Michael Matheny has no consulting income to report, and Frederic Resnic reports having modest consulting income from Boston Scientific Inc., Abbott Vascular Inc., Cordis Corp., and St. Jude Medical Inc., as well as research grants from The Medicines Company. Dr. Normand receives minor consulting income from the Medicines Company and Analytica International.

## Authors' contributions

MEM participated in the conception and design of the study, analysis and interpretation of the data, drafting of the manuscript, and critical revision of the manuscript. STN participated in the conception and design of the study, acquisition of the data, analysis and interpretation of the data, and critical revision of the manuscript. TPG, DMD, NLB, and SD participated in the conception and design of the study and critical revision of the manuscript. VDV participated in analysis and interpretation of the data and critical revision of the manuscript. FSR participated in the conception and design of the study, analysis and interpretation of the data, and critical revision of the manuscript. All authors read and approved the final manuscript.

## Pre-publication history

The pre-publication history for this paper can be accessed here:

http://www.biomedcentral.com/1472-6947/11/75/prepub
